# Low vision aids provision in an urban setting in Germany between 2014 and 2017: a regional population based study with healthcare claims data

**DOI:** 10.1007/s00417-024-06541-7

**Published:** 2024-06-18

**Authors:** M. L. Stolwijk, I. Meyer, S. L. van der Pas, J. W.R. Twisk, R. M.A. van Nispen, G. H.M.B. van Rens

**Affiliations:** 1 Amsterdam UMC, Vrije Universiteit Amsterdam, Ophthalmology, Amsterdam, The Netherlands; 2 Amsterdam Public Health, Quality of Care, Amsterdam, The Netherlands; 3 PMV Research Group, Faculty of Medicine and University Hospital Cologne, University of Cologne, Cologne, Germany; 4 Amsterdam UMC, Vrije Universiteit, Epidemiology and Data Science, Amsterdam, The Netherlands; 5 Amsterdam Public Health, Methodology, Amsterdam, The Netherlands

**Keywords:** Low vision aids, Low vision rehabilitation, Healthcare claims data, Visual impairment

## Abstract

**Purpose:**

Little is known about the utilization of low vision services (LVS) in Germany. To understand which persons and how often these services would be utilized, this study aimed to investigate low vision aids (LVAs) provision in an urban setting and to describe user characteristics and trends in their characteristics.

**Methods:**

A retrospective study based on a population-based healthcare claims database in Cologne (N = ~ 500,000), Germany. The study population comprised individuals, who were continuously insured at four large statutory health insurers and who redeemed a prescription for visual aids or aids for blindness between January 2014 and December 2017. We examined their socio-demographic and clinical characteristics. Trends in characteristics were examined with logistic and linear regression models over time.

**Results:**

Out of ~ 500,000 persons, 781 unique individuals (~ 0.2%) redeemed an LVA prescription. They were mainly female (68.7%), 60 years or older (75.3%) and had macular degeneration (50.6%) and/or glaucoma (25.9%). In the working-age subgroup, 33.8% were employed. Visual aids were most often prescribed (74.1%) and of all types of LVAs, individuals most commonly redeemed a prescription for magnifiers (35.8%), screen readers (34.3%) and/or canes (17.1%). Of the entire study population, 75.4% received their prescription from an ophthalmologist, 5.3% from a general practitioner and 7.1% from other medical specialists. Significant trends in characteristics of individuals who redeemed an LVA prescription were not found.

**Conclusions:**

Between 2014 and 2017, 781 individuals in Cologne redeemed an LVA prescription. They had characteristics which mostly can be explained by the epidemiology of VI. Results indicate that individuals that redeemed LVAs have a magnification requirement of ≥ 1.5-fold and ≥ 6-fold. Furthermore, next to ophthalmologists, general practitioners and other medical specialists seem to play a role in LVA provision as well, which should be taken into account by policy makers when planning interventions for increasing LVS provision. Our findings provide a starting point to examine LVS provision in Germany.

## Key messages

***What is known***

•Despite the relevance of low vision services (LVS), there are only a few studies on LVS provision in Germany. Furthermore, healthcare claims data have not been used before to describe LVS provision in Germany.

***What is new***

•We examined low vision aids (LVAs) provision in an urban setting in Germany based on population-based healthcare claims data spanning a four-year period.

•Our findings provide a starting point to examine LVS provision in Germany.

## Introduction

The most impactful consequence of ophthalmic diseases is visual impairment (VI), which is defined as low vision or blindness and is characterized by an irreversible loss of sight [1]. VI challenges the quality of life, due to impaired participation in daily life, increased risk of psychological distress, especially anxiety and depression, and increased risk of falls and fractures [2–5]. In addition, VI has a huge economic burden due to high healthcare costs and productivity losses [6].

In Germany, which has over 84 million inhabitants, between 500.000 and 1 million people are estimated to be visually impaired, of which the majority is caused by macular degeneration, glaucoma and diabetic retinopathy [7–10]. Most people affected are 60 years or older and with an ageing population, prevalence is expected to rise rapidly in the next decades [11]. Given this fact and its tremendous negative consequences, VI forms a great threat for public health and society.

Low vision services (LVS) are important to counteract this negative impact, as they have shown to be effective in improving quality of life and to be cost-effective from a societal perspective [12]. LVS in Germany are wide-ranging, from low vision aids prescription and training (e.g. magnifiers, electronic reading devices), to support and training in activities of daily living (e.g. cooking, dressing), training in mobility and orientation (e.g. walking with cane), and psychological therapy [13–15]. They teach individuals to compensate for their VI and to gain (back) independence.

LVS in Germany are offered in segmented form by different (healthcare) institutions, e.g. ophthalmology departments in hospitals/eye clinics or other medical practices, optician practices, social services/social work institutions and patient organizations. Professionals involved are ophthalmologists, opticians, specialized teachers for visually impaired, psychologists, but also volunteers of local patient organizations and other local societies for the visually impaired [13, 16, 17]. They are (partially) paid by health, retirement and accident insurance, by other integration assistance benefits for people with disabilities paid by the German government, or are free of charge as they are offered by local patient or charity organizations [13, 18]. Sometimes, however, patients must pay for it themselves.

Despite the available LVS facilities and the relevance of these services, there are only a few studies on LVS provision in Germany. International studies have shown that LVS delivery can be hampered by barriers, such as lack of referral by eye care professionals, distance to LVS and healthcare costs for services, leading to limited access for people who are in need of LVS and to inefficiency of service provision [19–24]. This could also be the case for Germany, but with the scarcity of research on LVS provision, little can be said about the adequacy of these services in this high-income country.

In the past two decades, there is a growing body of research that uses healthcare claims data to get insight into delivery of care and healthcare planning [25, 26]. With regard to LVS, recent studies have shown that population-based studies with healthcare claims data in countries where LVS is (partially) paid by health insurance, are valuable in gaining insight into trends in LVS utilization, characteristics of LVS users and possible barriers and facilitators [27]. Stolwijk et al. [28] found that LVS utilization had decreased in the Netherlands between 2015 and 2018, but showed an increase in certain LVS user characteristics such as physical comorbidity and utilization of low vision aids. Basilious et al. [27] conducted a regional study in Canada and found an increase of LVS utilization since 2009. However, barriers in service access were found with regard to age, sex and geographic location.

Against this background, this study provides an exploration of LVS provision and characteristics of LVS users in an urban setting in Germany based on regional healthcare claims data of the city of Cologne, North Rhine-Westphalia. LVS, which are (partially) funded by health insurance, comprise of low vision aids, occupational therapy and psychological therapy. As these therapies can also be provided outside the context of LVS, this study focuses on low vision aids (LVAs). The aim of the study was to investigate how many people received an LVA prescription and what kind of LVAs were prescribed between 2014 and 2017 in Cologne. In addition, we aimed to study socio-demographic- and clinical characteristics of individuals with an LVA prescription and trends in these characteristics in this period.

## Methods

### Design

We conducted a retrospective study based on a regional population-based healthcare claims database in Germany. The study was conducted in accordance with the ‘Good Epidemiological Practice’ by Hoffmann, Latza [29] and the ‘Good Practice of Secondary Data Analysis’ by the Working Group for the Collection and Use of Secondary Data (AGENS) of the German Society for Social Medicine and Prevention (DGSMP) and the German Society for Epidemiology (DGEpi) [30].

### The German health insurance system

Healthcare in Germany is paid by statutory and private health insurance (SHI, PHI) [31, 32]. The SHI is mandatory for all German citizens who have a gross annual income of less than 64,350 EUR (2022). Besides that, citizens who receive government benefits, e.g. employment benefit recipients, students, retired citizens, citizens who applied for pension, and certain family members are also insured by SHI. Citizens with a higher annual income can opt for PHI instead of SHI, but do not have to. This also applies to self-employees, freelancers, civil servants and some other groups. Both the SHI and the PHI charge 14.6% of the gross salary, which is equally shared between employer and employee. Furthermore, each health insurance charges an additional contribution to its members, which varies between 0.3% and 1.8% of the gross salary of the insured. Before 2021, citizens could switch insurance every 18 months, which was changed to 12 months after 2021.

Approximately 87% of German citizens are insured by SHI and around 11% have PHI. Among other benefits, the SHI covers treatment for disease, including inpatient and outpatient care, psychotherapy, dental care, nursing care at home, medical aids, socio-therapy and certain types of rehabilitative care.

### Data source

The healthcare claims data were requested from the Cologne Research and Development Network (CoRe-Net) [33]. The database contains data of the SHI of four big German health insurers (AOK Rheinland/Hamburg, BARMER Ersatzkrankenkasse (BARMER), DAK-Gesundheit (DAK) and pronova Betriebskrankenkassen (pronova-BKK)), including data of approximately 500.000 insured inhabitants of Cologne (~ 1 million) per year between 2014 and 2017.

### Study population

#### Low vision aids

In Germany, medical aids are generally covered if they are included in the list of medical aids by The Federal Joint Committee of Germany [34]. This list includes various product groups, to which available medical aids on the market are allocated with an individual product code. This study focused on LVAs that belong to product group ‘07’ (aids for blindness) and ‘25’ (visual aids). Our study focuses on 2014–2017 data. According to the medical aids list, insured individuals who were legally blind or who had severe visual impairment according to following definitions were eligible for funding of aids for blindness by health insurance in this period:

I. *Blind*: best corrected visual acuity of ≤ 0.02 in the better eye or an equivalent disturbance of vision (for example due to visual field loss);
II. *Severe visual impairment*: best corrected visual acuity of ≤ 0.05 but > 0.02 in the better eye or an equivalent disturbance of visual function. This applies when the impairment of vision results in a disability rating (GdS) of 100 according to the German law (§ 30 BVG) and blindness has not yet occurred.

Furthermore, the medical aids regulation of the German National Association of Statutory Health Insurance Funds defines which medical aids, among which visual aids, are covered by the SHI [35]. This study refers to the regulation that was valid between 2014 and 2017 [36–38]. According to this regulation, for people younger than 18 years old, all visual aids were funded. For people aged 18 years or older, visual aids were funded if they met following criteria:

I. *Visual impairment*: best corrected visual acuity of ≤ 0.30 or a binocular visual field of ≤ 10° around the central fixation point.

According to the medical aids regulation, visual aids need to be prescribed by an ophthalmologist. Aids for blindness can be prescribed by any medical specialist. This also applied to the 2014–2017 regulations.

### Sample selection

**Fig. 1:**
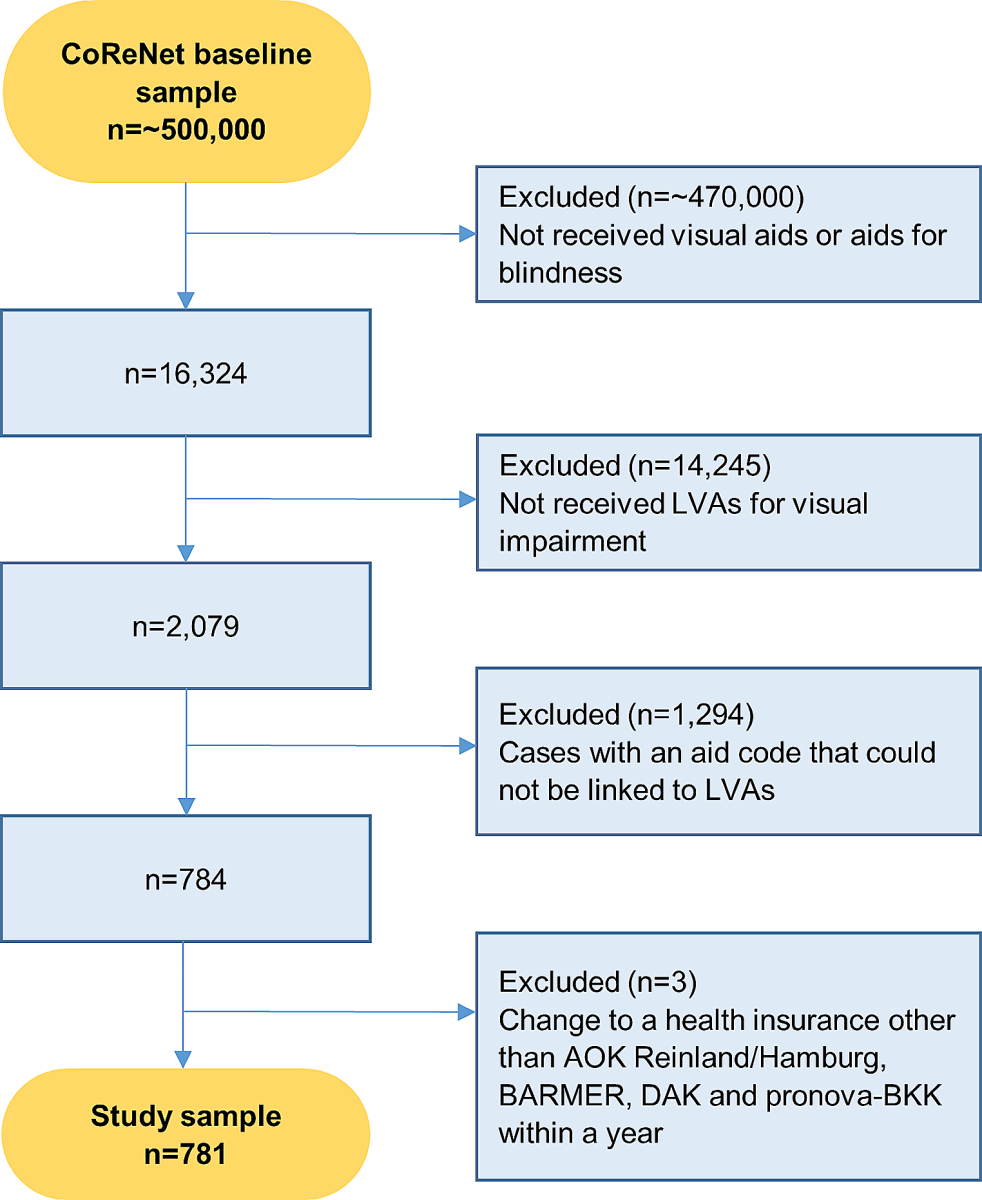
Flowchart of the study sample selection procedure Abbreviations: LVAs, Low Vision Aids

Figure 1 shows the results of the sample selection procedure. In this study, when we mention individuals who received a prescription or who received LVAs, we are referring to those who actually redeemed visual aids/aids for blindness/LVAs after receiving a prescription. Healthcare claims data of all insured individuals within the CoRe-Net database who received a prescription for visual aids and/or aids for blindness within the selected product groups at least once between the 1st of January 2014 until the 31st of December 2017 were requested for this study. This resulted in a CoRe-Net baseline dataset of *n* = 16,342.

For our research questions we selected those who actually received LVAs. Individuals who only received aids prescribed from the product group ‘visual aids’ not sufficient for people with VI, were excluded. These were individuals who received glasses and lenses, which are mainly prescribed for refractive error and astigmatism and therefore assumed not to be visually impaired by definition. This led to a sample size of n = 2,079.

Plausibility checks were done to retrieve false information in the claims data regarding the product codes within the selected product groups [29]. Of the 182 distinct product codes occurring in the dataset (*n* = 2,079), 44 codes could not be linked to the selected LVAs from the medical aids list. Of those, 26 were identified as pharmaceutical numbers and as not related to LVAs. For the remaining 18 missing codes, further plausibility checks were done to see if they could be linked to LVAs. For every year, we examined whether the prescriptions for these missing product codes were done by ophthalmologists and/or whether individuals with such a prescription were treated for ophthalmic diagnoses and/or with ophthalmic procedures within the inpatient care. For a missing product code to be considered as an LVA, we set following criterion: the prescription was done by an ophthalmologist and individuals with a prescription either had been treated for an ophthalmic disease leading to VI or had received an ophthalmic procedure. Specialist codes, codes of the International Classification of Diseases, 10th revision, German modification (ICD-10-GM codes) and medical procedure codes (German Uniform Assessment Standard, EBM) from the outpatient care within the data were used for this examination.

Eight out of the 18 remaining missing product codes were considered as LVAs, the other 10 remained missing for unknown reasons. All 36 missing product codes were excluded from the dataset, resulting in a remaining sample size of *n* = 784.

Furthermore, only continuously SHI insured individuals were included per year. More specifically, those who changed to an insurer other than the four included in the CoRe-Net were excluded from the dataset. The final study sample included 781 distinct individuals. The dataset contained individual annual socio-demographic data, including year of birth, sex, postal code, working status and death, as well as individual clinical data, including inpatient and outpatient patient history, containing ICD-10-GM diagnoses and clinical procedures.

### LVA prescriptions

We investigated the annual LVA prescriptions of the study population, the type of LVAs that were prescribed and the prescribing medical specialists of LVAs.

### Socio-demographic characteristics

The annual age and sex distribution, as well as working status of individuals who received LVAs were retrieved from the healthcare claims. We investigated whether individuals had an occupation or not in the year of their LVA prescription.

### Clinical characteristics

Clinical characteristics were investigated by looking at ophthalmic diagnoses, physical comorbidity and mental comorbidity based on ICD-10-GM codes from the inpatient and outpatient care. In Germany, outpatient care is most often provided outside the hospital in specialized medical care practices, such as ophthalmology clinics and general practitioner practices, whereas inpatient care is offered at hospitals.

We examined treatment prevalence of ophthalmic diagnoses in a stepwise approach to get insight into the diagnoses that led to VI and thus to prescription of LVAs to our study population. We first looked at the overall distribution of ophthalmic diagnoses within our study population. Therefore, we selected most certain ophthalmic ICD-10-GM codes (ICD codes H00–H59) from the inpatient and outpatient care, which are diagnoses coded as ‘confirmed’ within the outpatient care and diagnoses coded as ‘discharge diagnosis’ within the inpatient care. Next, we calculated the treatment prevalence for the diagnoses that most often occurred within our study population and that are most commonly related to VI. These comprised of diagnoses codes for macular degeneration, glaucoma, diabetic retinopathy and VI.

To minimalize the risk of false-positive diagnoses in the calculation of treatment prevalence, diagnoses need to be validated [39]. Commonly used internal validation strategies in studies with German healthcare claims data are the M2Q and the M1S criterion [40, 41]. M2Q stands for a minimum of two quarters and requires a coded diagnosis in at least two quarters within a year. This approach is commonly used to validate outpatient care diagnoses. M1S stands for a minimum of one quarter within the inpatient hospital sector (‘stationary’ care in Germany) and requires a coded discharge diagnosis in one quarter.

We calculated diagnosis prevalence of macular degeneration, glaucoma, diabetic retinopathy and VI by selecting individuals who had ≥ 1 discharge diagnosis within the inpatient care and/or ≥ 2 confirmed diagnoses in a minimum of two of four quarters (1-year period) within the outpatient care. Table 1 shows the diagnoses codes that were used for validation of the different ophthalmic diseases.

**Table 1:** Selection of ICD-10-GM codes for ophthalmic diagnosis validation

Ophthalmic diagnosis	ICD-10-GM diagnosis codes
Macular degeneration	H35.3
Glaucoma	H40.-, H40.0, H40.1, H40.2, H40.3, H40.4, H40.5, H40.6, H40.8, H40.9, H42.-, H42.0, H42.8
Diabetic retinopathy	H36.0, E10.3, E11.3, E12.3, E13.3, E14.3
Visual impairment	H54.0, H54.1, H54.2, H54.3, H54.4

For insights into physical comorbidity, we looked at the general distribution of comorbid physical diseases within the study population. We therefore selected certain ICD-10-GM chapters relating to physical comorbidity (Chap. 1–4, 6, 8–14). Having physical comorbidity was defined as having ≥ 1 registered ‘discharge diagnosis’ (inpatient care) and/or ≥ 2 ‘confirmed’ diagnoses (outpatient care) within at least one other of the selected ICD-10-GM chapters in a minimum of two of four quarters (1-year period) per year.

Mental comorbidity was investigated by looking at the distribution of diagnoses within Chap. 5 of the ICD-10-GM (Mental, Behavioral and Neurodevelopmental disorders) and the respective F-diagnoses. For defining mental comorbidity, the same diagnosis validation was applied to the relating F-diagnoses as for physical comorbidity. This also accounts for mental comorbidity at the level of the diagnosis.

### Statistical analyses

Descriptive statistics of the study population, as well as their socio-demographic and clinical characteristics were examined. Furthermore, trends in characteristics were investigated by defining the year of observation as independent variable and the different characteristics as dependent variable. For the analysis, most characteristics were dichotomized and a logistic regression was applied. Number of LVAs prescribed and age were defined as continuous dependent variables and were log-transformed due to a non-normal distribution, for these characteristics a linear regression analysis was conducted. The year 2014 was set as the reference and annual changes in the dependent variables in 2015 until 2017 were reported with respect to that year. There were missing values per year for some clinical characteristics, namely for ophthalmic diagnoses and physical and mental comorbidities. As the missing values were < 5%, they were not imputed for the analyses [42]. A Bonferroni correction for multiple testing was conducted by dividing the significance level of 0.05 by the number of models (16). Descriptive analyses were conducted with SQL programming language, regression analyses were conducted with the PROC LOGISTIC and the PROC REG procedure in SAS software version 9.4 (SAS Institute Inc., Cary, NC, USA).

## Results

### LVA prescription

Between 2014 and 2017, 781 (~ 0.2%) out of ~ 500,000 unique insured individuals received an LVA prescription. However, 12 individuals died during the study period (Table 2). LVA provision decreased by 19% between 2014 and 2015 (*n* = 217 in 2015 vs. *n* = 268 in 2014), increased by 13% between 2015 and 2016 (*n* = 245 in 2016 vs. *n* = 217 in 2015) and decreased again by 3% between 2016 and 2017 (*n* = 238 in 2017 vs. *n* = 245 in 2016). The majority of the study population (*N* = 781) received an LVA prescription in only one year (81.0%) with a mean annual number of prescribed LVAs of 1 (SD = 0.9). On average, individuals most often obtained a prescription of visual aids (74.1%). A mean of 28.3% received a prescription for aids for blindness. Of all types of LVAs, magnifiers (35.8%), screen readers (34.3%) and canes (17.1%) were most commonly prescribed. Furthermore, individuals mainly received their prescription by an ophthalmologist (75.4%). In an average of 5.3%, the prescription came from the general practitioner and in 7.1% from other medical specialists. Aids for blindness were mainly prescribed to individuals younger than 60 years old compared to individuals aged 60 years and older (15.5% vs. 12.8%, Table 3). However, this was not a large difference. With respect to visual aids the opposite could be observed (64.3% ≥60 years vs. 9.8% <60 years). There were no significant trends with respect to characteristics relating to LVA prescription (Table 4).

### Socio-demographic characteristics

The study population was mainly female (68.7%), 60 years or older (75.3%),unemployed (91.5%) and 24.5% was of working-age (15 – <65 years), of which 33.8% was employed. No significant trends were found with respect to these socio-demographic characteristics.

### Clinical characteristics

Between 2014 and 2017, an average 97.2% of the study population were treated in outpatient and/or inpatient care and received an ophthalmic condition registered by a medical specialist, 90.3% obtained their diagnosis by an ophthalmologist. Most prevalent ophthalmic ICD diagnoses that related to VI were macular degeneration (50.6%) and/or glaucoma (25.9%), 77.2% had other eye diseases which they could have had in addition to the three most prevalent diagnoses. Furthermore, almost the entire study population had physical comorbidity (94.3%) and almost half had mental comorbidity (43.1%). No significant trends were found regarding ophthalmic diagnoses, physical comorbidity and mental comorbidity.

**Table 2:** Characteristics of the study population in 2014–2017 (*N* = 781)

		2014 *n* = 268	2015 *n* = 217	2016 *n* = 245	2017 *n* = 238	Mean (4 years)
		*n* (%)	*n* (%)	*n* (%)	*n* (%)	*N* %
*LVA prescriptions*						
**Type of low vision aids** ^**a**^						
**Aids for blindness**		71 (26.5)	68 (31.3)	64 (26.1)	71 (29.8)	68.5 (28.3)
Electronic systems for information processing and output		12 (4.5)	8 (3.7)	15 (6.1)	9 (3.8)	11.0 (4.5)
Canes		41 (15.3)	41 (18.9)	33 (13.5)	51 (21.4)	41.5 (17.1)
Other		26 (9.7)	21 (9.7)	25 (10.2)	18 (7.6)	22.5 (9.3)
**Visual aids**		206 (76.9)	155 (71.4)	187 (76.3)	169 (71.0)	179.3 (74.1)
Magnifiers		89 (33.2)	78 (35.9)	97 (39.6)	83 (34.9)	86.8 (35.8)
Screen readers		100 (37.3)	66 (30.4)	86 (35.1)	80 (33.6)	83.0 (34.3)
Other		28 (10.4)	24 (11.1)	13 (5.3)	10 (4.2)	18.8 (7.7)
**Number of prescribed low vision aids** (***N*** = **1281**)		361 (28.2)	283 (22.1)	305 (23.8)	332 (25.9)	320.3 (25.0)
**Number of prescribed low vision aids per person, range 1–15, mean (SD)**		1 (0.6)	1 (1.3)	1 (0.8)	1 (0.8)	1 (0.9)
**Prescribing medical specialist** ^**b**^						
Missing		48 (17.9)	34 (15.7)	38 (15.5)	34 (14.3)	38.5 (15.9)
Ophthalmologist		201 (75.0)	164 (75.6)	187 (76.3)	178 (74.8)	182.5 (75.4)
General pracitioner		9 (3.4)	15 (6.9)	11 (4.5)	16 (6.7)	12.8 (5.3)
Other		18 (6.7)	11 (5.1)	18 (7.3)	22 (9.2)	17.3 (7.1)
*Socio-demographic characteristics*						
**Sex, female**		178 (66.4)	150 (69.1)	174 (71.0)	163 (68.5)	166.25 (68.7)
**Age, y, range 5-105, mean (SD)**		70 (21.7)	67 (22.7)	72 (22.2)	70 (23.3)	69.8
**Age group**						
0–14		7 (2.6)	6 (2.8)	13 (5.3)	12 (5.0)	9.5 (3.9)
15–34		17 (6.3)	19 (8.7)	5 (2.0)	14 (5.9)	13.8 (5.7)
35–49		20 (7.5)	20 (9.2)	12 (4.9)	13 (5.5)	16.3 (6.7)
50–59		24 (9.0)	21 (9.7)	19 (7.8)	17 (7.1)	20.3 (8.4)
60–69		23 (8.6)	19 (8.7)	21 (8.6)	20 (8.4)	20.8 (8.5)
70–79		46 (17.2)	44 (20.3)	46 (18.8)	44 (18.5)	45.0 (18.6)
80+		131 (48.9)	88 (40.5)	129 (52.7)	118 (49.6)	116.5 (48.1)
*≥60*		200 (74.6)	151 (69.6)	196 (80.0)	182 (76.5)	182.3 (75.3)
*<60*		68 (25.4)	66 (30.4)	49 (20.0)	56 (23.5)	59.8 (24.7)
**Working status**						
**Working (Age range, y**, **23–79)**		28 (10.4)	24 (11.1)	15 (10.3)	15 (6.3)	9.5 (8.5)
**Not working (Age range, y, 5–105)**		240 (89.6)	193 (88.9)	230 (93.9)	223 (93.7)	221.5 (91.5)
**Working-age (Age range, y, 15 – <65)**		69 (25.7)	67 (30.9)	49 (20.0)	52 (21.8)	59.3 (24.5)
Working		27 (39.1)	24 (35.8)	15 (30.6)	14 (26.9)	20.0 (33.8)
Not working		42 (60.9)	43 (64.2)	34 (69.4)	38 (73.1)	39.3 (66.2)
*Clinical characteristics*						
**Ophthalmic diagnoses** ^c^		264 (98.5)	209 (96.3)	233 (95.1)	235 (98.7)	235.3 (97.2)
Missing		4 (1.5)	8 (3.7)	12 (4.9)	3 (1.3)	6.8 (2.8)
Macular degeneration		139 (51.9)	99 (45.6)	129 (52.7)	123 (51.7)	122.5 (50.6)
Glaucoma		76 (28.4)	56 (25.8)	60 (24.5)	59 (24.8)	62.8 (25.9)
Diabetic retinopathy		31 (11.6)	14 (6.5)	20 (8.2)	8 (3.4)	18.3 (7.5)
VI		67 (25.0)	60 (27.6)	64 (26.1)	60 (25.2)	62.8 (25.9)
Other		213 (79.5)	175 (80.5)	189 (77.1)	170 (71.4)	186.8 (77.2)
Diagnosis by Ophthalmologist^d^		245 (91.4)	192 (91.9)	218 (93.6)	219 (93.2)	218.5 (90.3)
**Physical comorbidity**		253 (94.4)	204 (94.0)	229 (93.47)	227 (95.4)	228.3 (94.3)
**Mental comorbidity**		121 (45.1)	89 (41.0)	107 (43.7)	100 (42.0)	104.3 (43.1)
Mood (affective) Disorders^e^		56 (46.3)	45 (50.6)	56 (52.3)	53 (53.0)	52.5 (50.4)
Anxiety, dissociative, stress- related, somatoform and other nonpsychotic mental disorders^e^		49 (40.5)	35 (39.3)	40 (37.4)	37 (37.0)	40.25 (38.6)
Mental disorders due to known physiological conditions^e^		29 (24.0)	13 (14.6)	21 (19.6)	19 (19.0)	20.5 (19.7)
Other^e^		30 (24.8)	29 (32.6)	39 (36.4)	27 (27.0)	31.3 (30.0)

Data are n/n (%) or n/N (%), unless otherwise specified

Abbreviation: LVA, low vision aids. VI, visual impairment. SD, standard deviation

^a^ Individuals could have had a prescription for more than one type of low vision aid per year and could have a prescription in more than one year

^b^ The number of prescribing medical specialists relative to the number of individuals. Individuals could have received both visual aids and aids for blindness, and these low vision aids could have been prescribed by different healthcare providers

^c^ Number of individuals that received a ‘confirmed’ ophthalmic diagnosis within the outpatient care or a ‘discharge diagnosis’ within the inpatient care. Each diagnosis is dichotomous (yes/no). Individuals could have been treated for more than one ophthalmic condition per year

^d^ Number of individuals that received their ophthalmic diagnosis by an ophthalmologist

^e^ Data are n/121 (%), n/89 (%), n/107 (%), n/100 (%) for the years 2014–2017 for individuals who received an LVA prescription and a mental comorbidity. Each diagnosis is dichotomous (yes/no). Individuals could have been treated for multiple mental disorders

**Table 3:** Distribution of type of low vision aids prescribed by age and by sex in 2014–2017 (*N* = 781)

	2014 *n* = 268		2015 *n* = 217		2016 *n* = 245		2017 *n* = 238		Mean (4 years)
	n	%	n	%	n	%	n	%	
**Age**									
**Aids for blindness** ^a^									
<60	41	15.3	38	17.5	28	11.4	43	18.1	37.5 (15.5)
≥60	30	12.6	30	13.8	36	14.7	28	11.8	31.0 (12.8)
**Visual aids** ^a^									
<60	28	10.4	32	14.7	22	9.0	13	5.5	23.8 (9.8)
≥60	178	66.4	123	56.7	165	67.3	156	65.5	155.5 (64.3)
**Sex**									
**Aids for blindness** ^a^									
Female	44	16.4	45	20.7	36	14.7	36	15.1	40.3 (16.6)
Male	27	10.1	23	10.6	28	11.4	35	14.7	28.3 (11.7)
**Visual aids** ^a^									
Female	140	52.2	109	50.2	142	58.0	129	54.2	130.0 (53.7)
Male	66	24.6	46	21.2	45	18.4	40	16.8	49.3 (20.3)

^a^ Patients could have had a prescription for both, aids for blindness and visual aids per year

**Table 4:** Results of logistic and linear regression analysis: Effects of time (year) of characteristics of the study population (*N* = 781)

Outcome variables	2015^a^		2016^a^		2017^a^	
	OR [95%]	p*	OR [95%]	p*	OR [95%]	p*
		*LVA provision*				
**Type of low vision aids**						
Aids for blindness^b^	1.27 [0.85–1.88]	0.26	0.98 [0.66–1.45]	0.80	1.18 [0.90–1.74]	0.57
Visual aids^b^	0.75 [0.50–1.13]	0.32	0.97 [0.65–1.46]	0.33	0.74 [0.50–1.10]	0.23
**Number of prescribed low vision aids per person** ^†^	-0.03 [-0.10–0.04]	0.44	-0.05 [-0.12–0.01]	0.12	-0.01 [-0.09–0.05]	0.60
**Prescribing medical specialist**						
Ophthalmologist ^c^	1.03 [0.68–1.56]	0.95	1.08 [0.72–1.61]	0.71	0.99 [0.66–1.48]	0.79
General practitioner ^c^	2.14 [0.92–4.98]	0.19	1.35 [0.55–3.32]	0.58	2.07 [0.90–4.79]	0.23
Other^c^	0.74 [0.34–1.60]	0.19	1.10 [0.56–2.17]	0.78	1.42 [0.74–2.71]	0.13
		*Socio-demographic characteristics*				
**Sex (Female)**	1.13 [0.77–1.66]	0.90	1.24 [0.85–1.80]	0.32	1.10 [0.78–1.60]	0.91
**Age** ^†^	-0.05 [-0.14–0.05]	0.33	0.01 [-0.08–0.11]	0.78	-0.01 [-0.11–0.08]	0.76
**Working (not working)**	1.07 [0.60–1.90]	0.08	0.56 [0.29–1.07]	0.16	0.58 [0.30–1.11]	0.20
		*Clinical characteristics*				
**Ophthalmic diagnosis**						
Macular degeneration^d^	0.78 [0.54–1.12]	0.10	1.03 [0.73–1.46]	0.42	1.99 [0.70–1.41]	0.66
Glaucoma^d^	0.88 [0.59 − 1.32]	0.99	0.82 [0.55–1.22]	0.58	0.83 [0.56–1.24]	0.67
Diabetic retinopathy^d^	0.53 [0.27–1.02]	0.83	0.68 [0.38–1.23]	0.35	0.27 [0.20–0.59]	0.01
Visual impairment^d^	1.15 [0.76–1.72]	0.51	1.06 [0.71–1.58]	0.95	1.01 [0.68–1.51]	0.75
Other^d^	1.08 [0.68–1.69]	0.17	0.87 [0.57–1.33]	0.92	0.65 [0.43–0.97]	0.01
**Physical comorbidity** ^**d**^	0.93 [0.43–2.00]	0.80	0.85 [0.41–1.76]	0.50	1.22 [0.55–2.72]	0.42
**Mental comorbidity** ^**d**^	0.85 [0.59–1.21]	0.50	0.94 [0.67–1.34]	0.79	0.88 [0.62–1.25]	0.73

Reference group: ^a^2015; ^b^no prescription for this type of low vision aid; ^c^no prescription by this type of medical specialist, ^d^no diagnosis for this type of diagnosis

*Reported *p*-values are corrected. Bold is significant at *p* < 0.003 (i.e. after Bonferroni correction)

†regression coefficients are obtained from the linear regression analysis

## Discussion

This retrospective study aimed to investigate LVA provision in an urban setting in Germany. Visual aids were the most often prescribed type of LVAs and next to ophthalmologists, general practitioners and other medical specialists seemed to have played a role in LVA provision as well. Although we found some annual fluctuations in characteristics of individuals with an LVA prescription, no significant trends were found.

Our study showed that 781 individuals received an LVA prescription between 2014 and 2017 of a population of approximately 500.000 with an SHI at one of four large insurers in Cologne. In Germany, the estimated prevalence of having a VI in the general population is between 0.6 and 1.2%. For Cologne this means that approximately 6,500 to 13,000 persons have a VI based on 1,084,000 inhabitants. Extrapolating the number of LVAs that was prescribed to the overall population of Cologne yields an estimated 1,562 individuals with an LVA prescription in the four-year period (or *N* = 390 LVAs per year). Considering the estimated number of persons with VI in Cologne, the number of LVAs that were actually prescribed seems rather low [9, 10, 43]. One possible explanation can be that prescriptions occur at longer time intervals than could be covered by the data used. Another explanation could be that patients paid for the low vision aids themselves or already had an LVA before 2014–2017. However, it might also indicate there is a need for information provision about funding of LVAs by health insurance companies or healthcare providers. Future studies should further investigate LVA provision by starting off with a sample of people with a VI, as this information was not available in our data. Furthermore, there were annual fluctuations with respect to the number of people that received an LVA prescription, but no stable trend could be observed.

Of the investigated LVAs, visual aids were most often prescribed and in accordance with previous studies, screen readers and magnifiers were the most frequently prescribed types of visual aids, which indicates a magnification requirement of individuals with an LVA prescription of ≥ 1.5-fold for magnifiers and ≥ 6-fold for screen readers, respectively [36–38, 44–46].

Moreover, there were differences in provision between aids for blindness and visual aids with respect to age. Where the former where mostly prescribed to individuals younger than 60, visual aids were mostly prescribed to individuals aged 60 and older. An explanation could be that in older individuals, the eye disease resulting in VI might be more progressed and consequently, aids for blindness may be prescribed more frequently. As we had no insight into severity of the VI, more research is needed to examine the provision of type of LVAs by severity of the VI.

Individuals who received an LVA prescription were mainly female, 60 years and older and had macular degeneration and/or glaucoma. These socio-demographic characteristics can be explained by the epidemiology of VI in Germany [7]. Besides that, of the individuals who received an LVA and were of working-age, only 34% were employed. This is similar to employment rates of people with visual impairment in other high-income countries and is lower compared to the employment rate of 77% in the general working-age population in Germany, indicating reduced work participation of people with visual impairment, as found in other studies [47–50].

Our results further revealed that the study population had a high prevalence of physical comorbidity (94%) and mental comorbidity (43%). This might partly be explained by the definitions we used to investigate comorbidities, which might have caused an overestimation. However, studies have shown that both mental and physical comorbidity in people with a VI are common, which our results seem to confirm [4, 51]. Furthermore, our study population was mainly 60 years or older (75%). As the prevalence of physical comorbidities increases with age, older adults often have multiple physical comorbidities, which explains our high numbers as well [52].

Moreover, results indicate that, next to ophthalmologists, general practitioners and other medical specialists play a role in LVA provision as well. Although in the majority of the study population, LVAs were provided by ophthalmologists, some individuals received their prescription for both visual aids and aids for blindness by a general practitioner or other medical specialists. This is finding is plausible for aids for blindness, but not for visual aids, as for the latter the guideline for medical aids requires a prescription by an ophthalmologist. This discrepancy might be attributed to coding errors in the claims data, or instances were medical specialists deviated from the guideline. For example in patients for whom visiting their general practitioner or other medical specialist is easier, e.g. due to impaired mobility, compared to visiting their ophthalmologist.

### Strengths and limitations

To the best of our knowledge, this is the first study examining LVA provision in an urban setting in Germany based on population-based healthcare claims data. A strength of our study is that we were able to examine LVA provision over a four-year period using a population-based sample that included people insured at four large insurers, thereby enhancing representativeness of the findings.

However, by only including SHI and not PHI insurers and only four of the 113 to 123 SHI insurers in 2014–2017, our results may have been affected by selection bias and therefore reduced representativeness. For example, research has found differences regarding education level and socio-economic status between different SHI insurers [53].

Besides that, this study only represents reimbursable LVA provision. LVAs that are not funded by health insurers were not included.

As insurance claims data were not designed for research, but for billing purposes, potential coding errors and invalidity are well known challenges in research based on this type of data [39]. This might have affected our results. To reduce possible bias, we performed plausibility checks and validated diagnoses internally. However, the ophthalmic diagnoses we investigated in our study have not been validated on German claims data by other studies and we therefore had no reference. More research on this topic is warranted to assess the validity of our results.

Moreover, as in claims data no information is available on visual acuity, visual field defects and severity of the VI, we could not examine these parameters in our analyses.

Lastly, this study reflects the LVA provision context of Cologne, a large city in the west of Germany. As there are great differences regarding healthcare provision between rural and urban areas, results should cautiously be applied to other German healthcare contexts. This also accounts for translation of the study results to other countries, as LVA prescription guidelines and healthcare funding regulations differ largely internationally.

## Conclusion

Between 2014 and 2017, 781 individuals received an LVA prescription. They had characteristics which mostly can be explained by the epidemiology of VI. Results indicate that individuals that received LVAs have a magnification requirement of ≥ 1.5-fold and ≥ 6-fold. Furthermore, results indicate that besides ophthalmologists, general practitioners and other medical specialists seem to play a role in LVA provision as well, which should be taken into account by policy makers when planning interventions for increasing LVS provision. Our findings provide a starting point to examine LVS provision in Germany. Future studies should investigate LVA provision among people with a VI in Germany in different urban and rural settings, and examine differences between those who get an LVA prescription and redeem it and those who do not. This way insights into possible differences and inequalities may become clear. Finally, it would be valuable to include other types of LVS, such as psychological therapy or support and training in activities of daily living, as well as additional forms of funding to get a more comprehensive understanding of LVS provision in Germany and its adequacy.
